# Pompe Disease Could Mimic Exam Findings of Amyloidosis: Two Rare Diagnoses Bona Fide

**DOI:** 10.1155/2018/9615834

**Published:** 2018-10-28

**Authors:** Jithma P. Abeykoon, Narjust Duma, Jennifer A. Tracy, Margherita Milone, Ronald Go

**Affiliations:** ^1^Department of Internal Medicine, Mayo Clinic, Rochester, MN, USA; ^2^Division of Hematology, Mayo Clinic, Rochester, MN, USA; ^3^Department of Neurology, Mayo Clinic, Rochester, MN, USA

## Abstract

A 70-year-old female presented with a three-year history of evolving macroglossia causing dysphagia and dysarthria, with proximal muscle weakness. Given the classic physical finding of macroglossia, the patient underwent extensive evaluation for amyloidosis which proved to be negative apart from a bone marrow biopsy which stained positive for transthyretin without amino acid sequence abnormality, thus giving wild-type transthyretin amyloidosis. Since the wild-type transthyretin amyloidosis could not entirely explain her clinical presentation and evaluation, further studies were conducted in a sequential manner, thus leading to a diagnosis of Pompe disease explaining her presenting signs and symptoms including her macroglossia. Through this fascinating case, we attempt to highlight the approach for the diagnoses of two rare diseases in a patient by emphasizing the importance of having a broad differential diagnosis when presented with findings which may have been thought as pathognomonic for certain diseases.

## 1. Introduction

Pompe disease, also known as acid maltase deficiency, first described by the Dutch pathologist J. C. Pompe in 1932, was the first glycogen storage disease to be identified and occurs due to an autosomal recessive (AR) mutation leading to acid maltase also called acid alpha-glucosidase (GAA) deficiency. Acid maltase deficiency leads to accumulation of glycogen in lysosomes and cytoplasm resulting in tissue destruction. Based on a study done in the Netherlands, the two forms of Pompe disease, infantile and juvenile/adult, carry a prevalence of 1 in 138,000 and 1 in 57,000 people, respectively [1, 2]. Clinical presentation of late-onset Pompe disease could be heterogeneous owing to its involvement of multiple systems such as nervous, musculoskeletal, pulmonary, cardiac, and gastrointestinal, thus making a punctual diagnosis challenging to clinicians.

Wild-type transthyretin amyloidosis is also a rare disease. While the deposition of wild-type transthyretin in cardiac biopsies on autopsy has been noted in 25–30% of patients in the 7^th^ and 8^th^ decades, histologically significant moderate-to-severe amyloid deposition occurs about 5% and a very small number of patients manifest clinical symptoms [3, 4]. By highlighting disease presentation, approach to clinical diagnosis, and diagnostic evaluations, we present a fascinating case where these two rare diseases were diagnosed simultaneously in one individual and also highlight the importance of having a broad differential diagnosis when patients present with clinical signs which some may consider as pathognomonic for certain diseases.

## 2. Case Presentation

A 70-year-old female presented to the clinic reporting a three-year history of progressive symptoms initially with increased salivation, followed by slurred speech and dysphagia as well as macroglossia and hypogeusia. During further questioning, she reported a frequent “choking sensation” while eating and food regurgitations. She also noticed progressive lower extremity weakness over the same duration but denied falls or gait instability. She denied dysgeusia, odynophagia, anosmia, diplopia, bowel or bladder incontinence, or other neurological symptoms.

Her past medical history was significant for gastroesophageal reflux disease controlled with omeprazole, right breast adenocarcinoma without sentinel lymph node involvement, status after mastectomy treated with tamoxifen, and idiopathic right hemidiaphragmatic paralysis diagnosed 25  years ago. Prior surgical history apart from mastectomy was significant for a sacral colpopexy, hysterectomy, and right carpal tunnel release.

She was initially evaluated by her primary care physician; initial testing included a magnetic resonance imaging (MRI) scan of the brain done to rule out a cerebrovascular event which was negative for vascular abnormalities or ischemia. Due to the progressive nature of macroglossia, lower extremity weakness, and dysphagia, clinicians initially suspected myasthenia gravis. On further evaluations, acetylcholine receptor-binding antibodies were negative, and the patient did not improve after a trial of pyridostigmine and intravenous immunoglobulin. She also underwent a lumbar puncture, yielding normal results.

The patient continued to undergo multiple procedures, all nondiagnostic until she presented to our institution. At that time, her symptoms continued to worsen, significantly affecting her quality of life and causing weight loss due to the inability to move the food bolus forward due to the size of her tongue. On physical exam, no neuromuscular abnormalities were observed. The cranial nerves examination was significant for moderate-to-severe tongue weakness with tongue deviation to the right and associated macroglossia. Motor examination revealed normal strength of the neck flexors and extensors, mild-to-moderate weakness of the external rotators of the upper extremities and pectoral muscles bilaterally, mild weakness of the deltoid and biceps bilaterally with spared triceps and brachioradialis, and mild weakness of interossei and hypothenar bilaterally with spared thenar muscles. In the lower extremities, she had moderate weakness of the iliopsoas, moderate-to-severe weakness of the gluteus medius bilaterally (right weaker than left), and normal strength of the quadriceps, hamstrings, calf, and tibialis anterior bilaterally. She was able to get up from the chair without support. She had an asymmetric, waddling gait and was able to walk on toes. Tendon reflexes were normal with the exception of decreased ankle reflexes, +1 when +2 being normal. She had no clinical myotonia.

She was initially seen by neurology; an electromyogram revealed a myotonic myopathy with electrophysiological features supportive of inflammation, fiber splitting, vacuolization, and/or myonecrosis. These findings in conjunction with macroglossia were thought to be secondary to amyloid or other inflammatory myopathy.

Laboratory evaluation revealed normal complete blood counts and differential, ferritin, folate, and vitamin B12 levels in serum. Immunophenotype of the plasma cells did not reveal any monoclonality, and serum and urine electrophoresis with immunofixation failed to reveal any monoclonal disease or abnormalities associated with free light chains. The electrolyte panel was also within the normal limit with a creatinine clearance of 96 mL/min. The liver function test was within normal limits. Muscle enzymes were evaluated, and they revealed an increased creatinine kinase at 242 (normal: 38–176 U/L), lactate dehydrogenase at 297 (normal: 122–222 U/L), and aldolase level at 8.5 (normal: <7.7 U/L). Beta-2-microglobulin was within normal limits.

Due to history of breast cancer and with the goal of ruling any structural abnormalities, the patient underwent computed tomography (CT) and MRI of the head and neck which were notable for a soft tissue nodule at the base of the tongue and diffusely infiltrated fat signaling in the tongue as well as age-related atrophy of the cerebral white matter. The pulmonary function test showed mild-to-moderate restriction and reduced total lung capacity. Overnight pulse oximetry showed a gas exchange abnormality as well as significant sleep-disordered breathing. Echocardiogram revealed no evidence of cardiac amyloid and normal left ventricular chamber size and interventricular septum with an ejection fraction of 66%.

**Figure 1:**
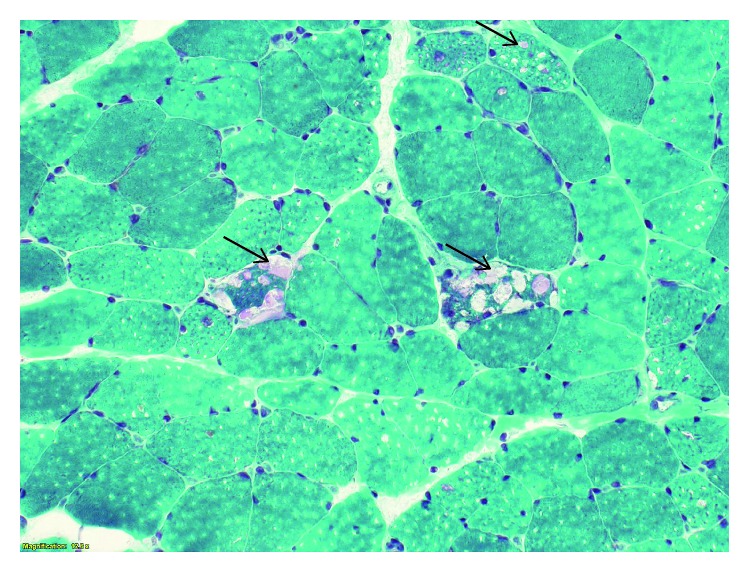
Trichrome stain showing fibers with multiple vacuoles (arrows). These are commonly seen in acid maltase deficiency and are associated with glycogen accumulation.

**Figure 2:**
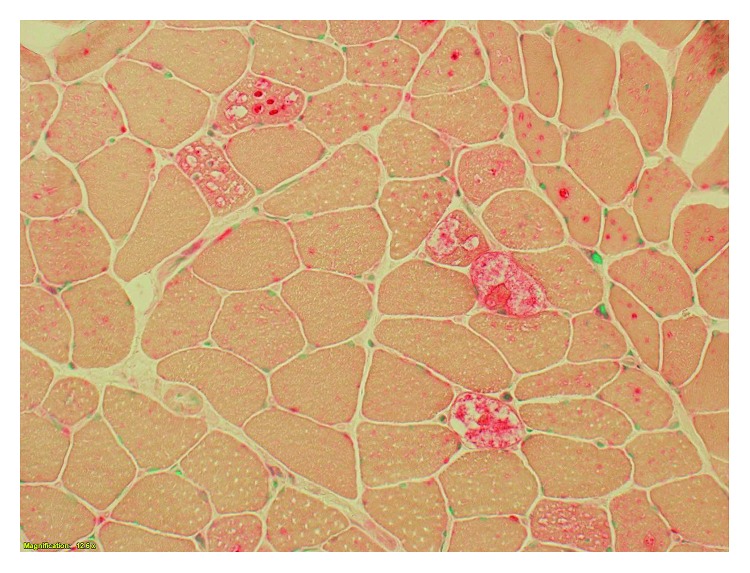
Acid phosphatase stain showing increased reactivity (appearing red) in many vacuolated and nonvacuolated fibers. Acid maltase is present in lysosomes, and the increased acid phosphatase reactivity is representative of lysosomal dysfunction.

After evaluation by hematology and otolaryngology, the decision was to proceed with a tongue biopsy which revealed hyperplastic changes with features suggestive of degeneration of the underlying skeletal muscle. This was negative for amyloid deposition. A pectoralis muscle biopsy showed increased acid phosphatase in many of the vacuolated fibers suggestive of a vacuolar myopathy, most consistent with acid maltase deficiency (Figures 1 and 2). Some structurally abnormal fibers had increased PAS-positive material. Congo red stained sections revealed no congophilic deposits. Abdominal fat aspirate was negative for amyloid deposition.

Due to the findings of the electromyogram (EMG) and pectoralis muscle biopsy blended with the clinical presentation, acid alpha-glucosidase activity in blood was evaluated, and it was revealed to be 0.0 (normal: >0.5 nmol/h/mL). Bone marrow biopsy was done to further evaluate any evidence of amyloidosis or other hematologic diseases and showed normocellular bone marrow with morphologically normal trilineage hematopoiesis. Focal amyloid deposition in the periosteum was identified, and liquid chromatography and tandem mass spectrometry revealed the amyloid to be transthyretin without amino acid sequence abnormality suggestive of age-related transthyretin amyloidosis (senile systemic amyloidosis).

The diagnosis of wild-type transthyretin amyloidosis by itself could not explain her entire clinical presentation and evaluations and prompted further clinical evaluations. Given the lack of acid alpha-glucosidase activity in blood, coupled with EMG and pectoralis muscle biopsy findings, and clinical signs and symptoms of lower extremity weakness, dysphagia, macroglossia, abnormal pulmonary function test, abnormal overnight oximetry, and elevated muscle enzymes suggested a diagnosis of glycogen storage disease. Hence, a DNA analysis of the white blood cells was done which showed heterozygote alteration of c.-32–13T>G (g.78078341) and c.1841C>A (g.78086463) genetic alteration causing to have Thr614Lys amino acid alteration, which is a pathogenic mutation that is typically associated with late-onset Pompe disease [5]. After confirming the genetic analysis, a diagnosis of adult-onset Pompe disease was made.

Since the patient's liver function was intact and amyloid deposition was only evident in the bone marrow with wild-type transthyretin deposition, the decision was to monitor for wild-type transthyretin amyloidosis without any treatment. For her late-onset Pompe disease, she was started on enzyme replacement therapy with biweekly intravenous alpha-glucosidase (20 mg/kg). After 8 months of follow-up, the patient reported that her dysphagia, slurred speech, lower extremity weakness, and walking distance have considerably improved as well as her general well-being. Her macroglossia remained unchanged, and her amyloidosis continues to be monitored by a local hematologist.

## 3. Discussion

Pompe disease is a neuromuscular disease associated with GAA deficiency which is an enzyme that hydrolyses alpha-1, 4- and alpha-1, 6-glucosidic linkages of glycogen in lysosomes under low pH environments. Deficiency of this enzyme causes accumulation of glycogen in lysosomes and cytoplasm causing cell destruction. The adult-onset disease presents mostly with pelvic and pectoral girdle muscular weakness and gastrointestinal symptoms such as difficulty in swallowing, gastroesophageal reflux, diarrhea, and constipation [6]. Diaphragmatic involvement causing respiratory distress and sleep-disordered breathing is also seen [6]. The diagnosis is made by assessing GAA enzyme levels in white blood cells and/or characteristic muscle biopsy findings, and gene sequencing is done to confirm the diagnosis [7].

In our patient, presenting symptoms of dysphagia and lower extremity weakness with elevated creatine kinase, lactate dehydrogenase, and aldolase raised the suspicion of Pompe disease. Abnormal overnight oximetry with pulmonary function tests and EMG findings of fiber splitting, vacuolization, or myonecrosis in the setting of diaphragmatic involvement further strengthened the suspicion. Assessment of the GAA activity level and genetic analysis confirmed the diagnosis.

The pathophysiology of wild-type transthyretin amyloidosis or senile systemic amyloidosis is associated with deposition of unmutated transthyretin protein leading to organ dysfunction. The prognosis is mainly related to cardiac involvement, and median overall survival of senile amyloidosis with cardiac involvement is about 75 months compared to patients with the most common type of amyloidosis, AL amyloidosis, with cardiac involvement which is about 11 months [8]. The diagnosis is made by detection of wild-type transthyretin protein in tissue as seen in the bone marrow periosteum in our patient.

Further, although macroglossia is a well-known clinical sign in the setting of AL amyloidosis, it could also be associated with other ailments as seen in our patient [9]. Congenital macroglossia could be seen in congenital disorders leading to vascular malformation, muscular enlargement such as Beckwith–Wiedemann syndrome, systemic disorders such as mucopolysaccharide storage disorders, and Pompe disease [9]. Acquired macroglossia could be seen in tumors such as lymphoma, epidermoids carcinoma, AL amyloidosis, acromegaly, and local reactive changes such as vascular congestion. Limited to one case report, familial transthyretin (ATTR) was also associated with macroglossia where biopsy of the tongue confirmed the diagnosis [10]. To our knowledge, there are no reported cases of macroglossia in patients with wild-type transthyretin amyloidosis. In our patient, the biopsy of the tongue was inconclusive with the diagnosis of wild-type transthyretin amyloidosis and the etiology of macroglossia was attributed to Pompe disease. As it is demonstrated in our patient, macroglossia is not a pathognomonic sign for amyloidosis, and one should have a broad differential diagnosis when encountered with a clinical sign as macroglossia, thus eliminating anchoring and availability bias. Further, absence of diagnosis of AL amyloidosis in a patient with macroglossia should not prevent the clinician from additional investigations to establish the etiology of this clinical sign.

Through this case report, we present two rare diagnoses. To our knowledge, this case is the first documentation of Pompe disease and wild-type transthyretin amyloidosis presenting simultaneously in one patient. Although the diagnosis of wild-type transthyretin in a 70-year-old patient could be an incidental finding, possible association of this disease with Pompe disease could exist and merit evaluation. Further, timely diagnosis of Pompe disease will improve patients' well-being as enzyme replacement therapy has shown to be promising in increasing muscle strength, walking distance, and reducing dysphagia and other disease specific symptoms as it was well depicted in our patient's response to therapy [11]. Thus, clinicians should be aware of this disease when there are suggestive presenting symptoms, so that punctual diagnosis could be made, making our patients the ultimate beneficiaries.
